# Modification of the AFM Sensor by a Precisely Regulated Air Stream to Increase Imaging Speed and Accuracy in the Contact Mode

**DOI:** 10.3390/s18082694

**Published:** 2018-08-16

**Authors:** Andrius Dzedzickis, Vytautas Bucinskas, Darius Viržonis, Nikolaj Sesok, Arturas Ulcinas, Igor Iljin, Ernestas Sutinys, Sigitas Petkevicius, Justinas Gargasas, Inga Morkvenaite-Vilkonciene

**Affiliations:** 1 Department of Mechatronics, Robotics, and Digital Manufacturing, Vilnius Gediminas Technical University, LT-03224 Vilnius, Lithuania; andrius.dzedzickis@vgtu.lt (A.D.); vytautas.bucinskas@vgtu.lt (V.B.); darius.virzonis@vgtu.lt (D.V.); nikolaj.sesok@vgtu.lt (N.S.); igor.iljin@vgtu.lt (I.I.); ernestas.sutinys@vgtu.lt (E.S.); sigitas.petkevicius@vgtu.lt (S.P.); justinas.gargasas@vgtu.lt (J.G.); 2 Panevėžys Competence Center of Technology and Business, Kaunas University of Technology, LT-37164 Panevėžys, Lithuania; 3 Department of Nanoengineering, Center for Physical Sciences and Technology, LT-02300 Vilnius, Lithuania; ulcinas@ftmc.lt; 4 Department of Electrochemical Material Science, Center for Physical Sciences and Technology, LT-02300 Vilnius, Lithuania

**Keywords:** atomic force microscopy, cantilever’s mathematical model, dynamic characteristics, nonlinear stiffness, high speed

## Abstract

Increasing the imaging rate of atomic force microscopy (AFM) without impairing of the imaging quality is a challenging task, since the increase in the scanning speed leads to a number of artifacts related to the limited mechanical bandwidth of the AFM components. One of these artifacts is the loss of contact between the probe tip and the sample. We propose to apply an additional nonlinear force on the upper surface of a cantilever, which will help to keep the tip and surface in contact. In practice, this force can be produced by the precisely regulated airflow. Such an improvement affects the AFM system dynamics, which were evaluated using a mathematical model that is presented in this paper. The model defines the relationships between the additional nonlinear force, the pressure of the applied air stream, and the initial air gap between the upper surface of the cantilever and the end of the air duct. It was found that the nonlinear force created by the stream of compressed air (aerodynamic force) prevents the contact loss caused by the high scanning speed or the higher surface roughness, thus maintaining stable contact between the probe and the surface. This improvement allows us to effectively increase the scanning speed by at least 10 times using a soft (spring constant of 0.2 N/m) cantilever by applying the air pressure of 40 Pa. If a stiff cantilever (spring constant of 40 N/m) is used, the potential of vertical deviation improvement is twice is large. This method is suitable for use with different types of AFM sensors and it can be implemented practically without essential changes in AFM sensor design.

## 1. Introduction

Atomic force microscopy (AFM) is widely used for measurements of various properties of materials, including surface topography, friction, adhesion, and viscoelasticity on an atomic scale [1,2]. The main limitation of AFM is low scanning speed, which depends on multiple dynamic factors, such as the bandwidth of the probe detection system [3], the positioning system [4], the probe type [5], and the probe and surface material [6], etc. Also, the data transfer and processing capabilities will limit the data acquisition speed [7]. Various approaches have been used to resolve this problem, such as: (i) designing a new type of actuator [8]; (ii) improving both the optical beam deflection and the electronic readout systems [3]; (iii) using a self-actuating high quality piezoelectric lead zirconate titanate (PZT) cantilever with piezo resistors [9]; (iv) using small cantilevers [10]; (v) using a high-resonant-frequency cantilever [11]; (vi) using high resonance frequency, thermally actuated piezo-resistive cantilevers [12]; (vii) utilizing a Q-controlled natural vibration shape of an AFM cantilever to perform the function of the actuator [13]; (viii) a control system approach [14]. All these ideas are based on the introduction of new devices to the sensory part or for improvement of the AFM control system. In general, solutions of this type could not be applied to AFMs of different configurations. Therefore, a more versatile and convenient AFM speed improvement system is needed.

Together with the limited bandwidth of the AFM scanner, one of the main factors limiting scanning speed is the dynamic behavior of the cantilever, causing the excitation of the higher modes of cantilever vibration, or complete loss of contact with the surface. The surface properties in contact mode AFM are revealed by observing the cantilever deflections caused by the tip–sample interaction around the initial set interaction force. Low initial interaction force allows for the scanning of soft and biological surfaces without damage. However low initial interaction force and high scanning speed can lead to contact loss between the probe and surface if the feedback system does not have sufficient bandwidth to maintain the constant interaction force. Increasing the initial interaction force is one possibility for resolving this issue; however, as the scanning speed and surface roughness increase, the interaction force needs to increase as well, reaching unacceptable levels, since in the case that the interaction force is higher than the Van der Waals repulsive force, the probe can be adhered onto a surface. A preferred solution would include adding the nonlinear spring, which exerts an increasing downward force on the probe. A new engineering solution, the idea of adding such a nonlinear spring without changing the hardware part of the sensor, was proposed in our earlier report [15]. The cantilever’s tip and surface interaction in such a system is controlled by an additional force, which is created by the air stream [16]. In the case where the cantilever’s tip loses contact with the surface at a high scanning speed, the air stream helps to keep the probe and surface in contact, due to the nonlinear controlled compressive force.

In the other hand, the air stream creates additional stiffness in the AFM sensor, and this should not create any additional force when the contact between the tip and the surface is stable. The air stream pressure and velocity are low; thus, its possibility to create a vibration of any kind, or interact with a hard sample surface, is far beyond limit. For the introduction of the airflow with the microscope, it is enough to produce a cantilever holder with an installed micro air duct and a precise airflow control system. The effect of an applied aerodynamic force depends on: the initial gas pressure, the air duct diameter and shape, and the initial gap size between air duct and cantilever surface [16,17].

The present research is aimed at developing a mathematical model for design of the AFM cantilever holder with an air duct.

## 2. Dynamic Model of the AFM Cantilever

**Figure 1:**
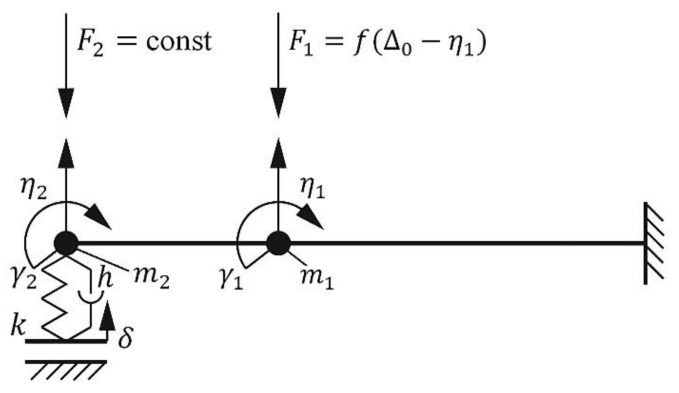
Dynamic model of the atomic force microscopy (AFM) mechanical sensor system.

Our research is focused on the modelling of the AFM cantilever, which is affected by air stream, in order to form a mathematic description of the dynamic response to kinematic excitation, which can be used to analyze the dynamic behavior of modified (with applied air stream, *F*_1_ ≠ 0) and non-modified (without air stream, *F*_1_ = 0) cantilevers (Figure 1).

**Figure 2:**
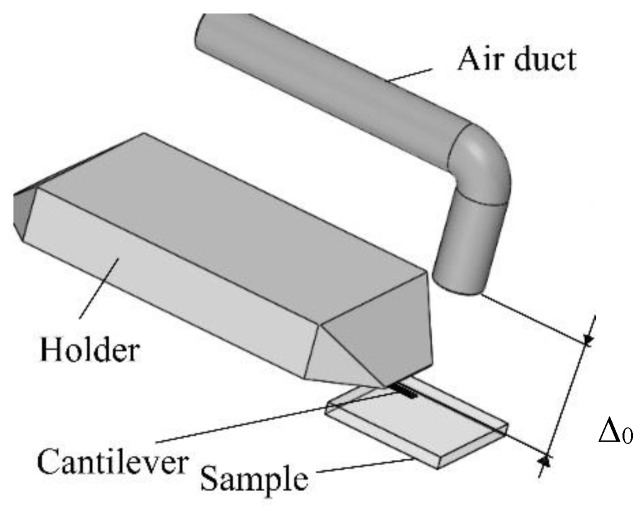
AFM sensor system, modified by implementing an air duct.

The cantilever, which can be assumed to be a rod with a rectangular cross-section, is approximated by two elastic elements with a concentrated mass at the ends. It is assumed that each element has a mass and a moment of inertia, which are used to evaluate the deflection and rotation angles of the rod’s end. In the lumped mass model, the mass of each element is transferred to the end point of the elastic element, as shown in Figure 1. It is assumed that the end point is shifted to approximately 25.5% of the element’s mass [18]. Therefore, the moment of the rod’s inertia around its attachment point is evaluated. Supposing that the cantilever is made from silicon nitride; the properties of this material correspond to the Kelvin-Voigt material model. The dynamic characteristics of the probe are approximately estimated as elastic elements with damping. The main system coordinates are *η*_1_, *γ*_1_, *η*_2_, and *γ*_2_, and they describe the linear and rotational displacements of the cantilever points of interest. The initial clamping force of the cantilever is approximated by a constant force *F*_2_, which acts in the negative coordinate *η*_2_ direction. The model also includes a nonlinear force *F*_1_, introduced by the airflow. This force depends on the gap size between the air supply tube and the cantilever surface (Figure 2):

(1) $$F_{1}=f\left(\Delta_{0}-\eta_{1}\right),$$

where Δ_0_—size of the initial gap between the end of the air duct and the cantilever upper surface, when the cantilever is not affected by air stream.

The roughness of the sample surface, which kinematically excites system oscillations [19], is described by coordinate *δ*. The probe is approximated by an elastic element with the coefficient of stiffness *k*, and damping with the coefficient of damping *h*. The elasticity of the cantilever is evaluated by the cross-section parameter *E* × *I_sk_*, where *E*—Young’s modulus, *I_sk_* = *w* × *t*^3^/12—moment of inertia of the cross-section of the beam with respect to the horizontal axis, where *w*—width of cantilever and *t*—thickness of cantilever. It is considered that the linear probe movements are described by coordinate *η*_2_. The mathematical model is created using Lagrange equations of the second type in the matrix form:

(2) $$\left[A\right]\left\{\ddot{q}\right\}+\left[B\right]\left\{\dot{q}\right\}+\left[C\right]\left\{q\right\}=\left\{Q\right\},$$

where [*A*]—matrix of inertia forces; [*B*]—matrix of damping coefficients; [*C*]—matrix of stiffness coefficients; {*q*} = {*η*_1_, *γ*_1_, *η*_2_, *γ*_2_}*^T^*—vector of generalized coordinates; {*Q*}—vector of generalized forces.

The derivations of elements of matrices [*A*], [*B*], [*C*] and vector {*Q*} are provided in Appendix A. The final system of equations with nonzero value of stiffness *k* and damping *h* was obtained by inserting those elements into the original equation:

(3) $$\left\{ \begin{array}{l} m{\;}_{1}\ddot{\eta }{\;}_{1}+B{\;}_{11}\;\dot{\eta }{\;}_{1}+B{\;}_{12}\;\dot{\gamma }{\;}_{1}+B_{13}{\dot{\eta }}_{2}+B_{14}{\dot{\gamma }}_{2}+C_{11}\eta_{1}+C_{12}\gamma_{1}+C_{13}\eta_{2}+C_{14}\gamma_{2}=-F_{1};\\ I_{1}{\ddot{\gamma }}_{1}+B_{21}{\dot{\eta }}_{1}+B_{22}{\dot{\gamma }}_{1}+B_{23}{\dot{\eta }}_{2}+B_{24}{\dot{\gamma }}_{2}+C_{21}\eta_{1}+C_{22}\gamma_{2}+C_{23}\eta_{2}+C_{24}\gamma_{2}=0;\\ m_{2}{\ddot{\eta }}_{2}+B_{31}{\dot{\eta }}_{1}+B_{32}{\dot{\gamma }}_{1}+\alpha \left(\frac{24EI_{sk}}{L_{2}^{3}}\right){\dot{\eta }}_{2}+h{\dot{\eta }}_{2}+B_{34}{\dot{\gamma }}_{2}+C_{31}\eta_{1}+C_{32}\gamma_{1}+\left(\frac{24EI_{sk}}{L_{2}^{3}}\right)\eta_{2}+\\ +k\eta_{2}+C_{34}\gamma_{2}=-F_{2}+k\delta +h\dot{\delta };\\ I_{2}{\ddot{\gamma }}_{2}+B_{41}{\dot{\eta }}_{1}+B_{42}{\dot{\gamma }}_{1}+B_{43}{\dot{\eta }}_{2}+B_{44}{\dot{\gamma }}_{2}+C_{41}\eta_{1}+C_{42}\gamma_{2}+C_{43}\eta_{2}+C_{44}\gamma_{2}=0. \end{array}\right.,$$

where *h* = *α k*.

**Figure 3:**
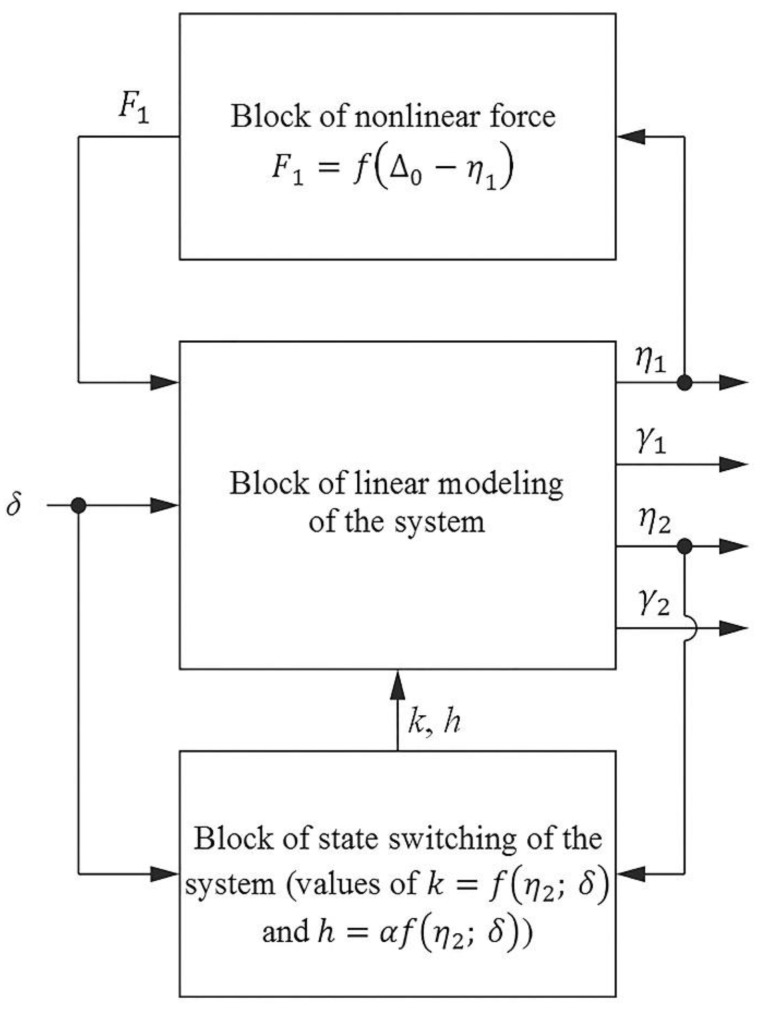
Schematic Simulink diagram of mathematical model: *δ*—coordinate of kinematic excitation, *η*_1_, *γ*_1_, *η*_2_, *γ*_2_—output coordinates.

In the other state of the system, the difference between the equations are in the values of *k* = 0 and *h* = 0. The equations are solved with respect to the second derivatives of generalized coordinates. For each equation, the corresponding structural diagram and the MatLab/Simulink model was built. All of these diagrams, representing the corresponding equations, are joined into the general structural diagram. Nonlinear elements in the equations are excluded into different blocks. The simplified structural schematic is presented in Figure 3.

The dynamic model of the AFM sensor consists of three structural blocks. The main block of the structural diagram represents the linear part of the system. The input of this block is the coordinate, depending on the roughness of the sample surface; the output is the generalized coordinates of beams. The force block represents a nonlinear force and additional stiffness. The state block controls the state of the additional force. This block controls the contact integrity and disconnects a contact stiffness when the contact force changes direction from a compressive force to a tensile one.

The parameters of the aerodynamic force were determined from the theoretical 3D model of the microscope cantilever, as reported previously [17]. This model describes the dependencies among the air gap, the pressure of compressed air, and the resulting force on the cantilever. The displacement of the cantilever at coordinate *η_2_* (Figure 1) defined from results of finite element modelling (FEM) analysis [17] and approximated by polynomial in respect to relative gap and expressed in terms of our model, is:

*η*_2_ = −0.0007·(Δ_0_ − *η*_1_)^3^ + 0.0033·(Δ_0_ − *η*_1_)^2^ + 0.0063·(Δ_0_ − *η*_1_) + 0.0049 (4)

Implementation of our model and obtaining realistic modelling results requires some assumptions and initial conditions. As the excitation of the tip of the cantilever is performed kinematically, there is a need to create displacement of the tip data in the time domain.

### Initial Assumptions for Modelling

**Figure 4:**
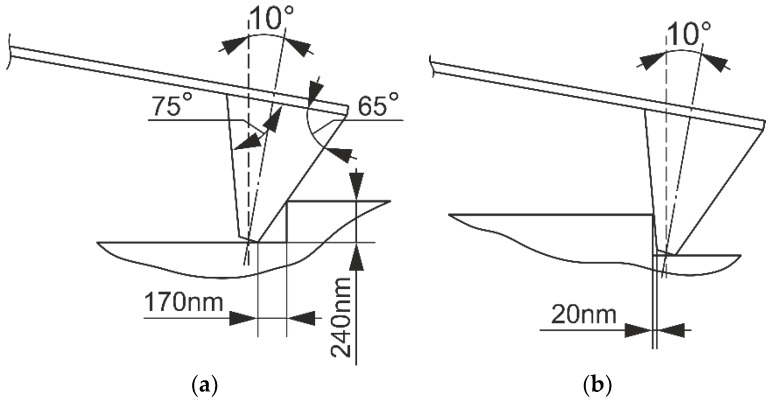
Interaction of the cantilever probe surface to the calibration grate: (**a**) front slope and (**b**) rear slope.

The model was excited kinematically using ideal rectangular shape input, which corresponds to the ideal case of the calibration grating (pitch 2 μm). Due to interaction between the cantilever probe to the sample surface, the ideal rectangular input generated alterations to real displacement of the cantilever. In this case, the input signal took into account the geometry of the cantilever probe; the angle between vertical sample surface and cantilever base was 10°. The cantilever tip displacement will be different when passing the front and back slopes, as shown in Figure 4.

**Figure 5:**
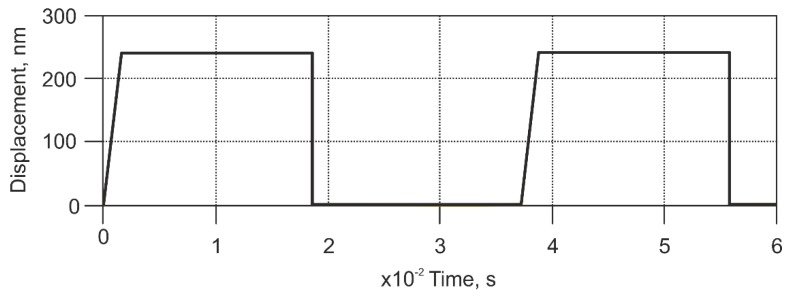
Real time domain kinematic input signal for a scanning speed of 112 μm/s from a square surface profile.

As seen from Figure 4, in the case when the cantilever approaches “plateaus”, the cantilever tip travels a distance of 170 nm in the horizontal direction until it reaches the top of the “plateaus”, in case then cantilever moves away from the “plateaus”, it will travel 20 nm until the tip reaches the bottom of the “hole”. Taking into account these relations and the pitch of the calibration grating, a dependency between the profile height and the horizontal distance (time) traveled by the cantilever tip was found. Therefore, the resulting model input signal distorts the square impulses. This was later obtained from kinematic relations input displacements transformed into time domains for each case of scanning speed; the particular case for a scanning speed of 112 μm/s is presented in Figure 5.

It is necessary to note that model provides the of the cantilever tip behavior during the scan process; a real AFM output is formed from the feedback of the resulting force control system.

## 3. Experimental Technique

### 3.1. Experimental Setup

**Figure 6:**
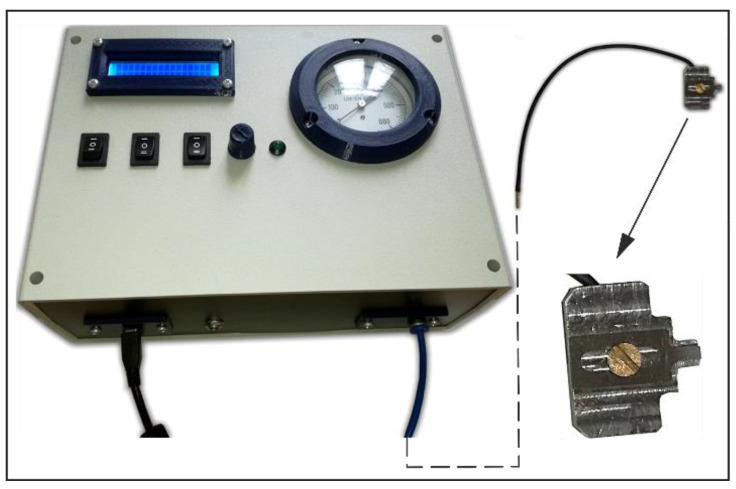
Air supply system.

An AFM cantilever was attached to the base of a digital versatile disk (DVD) optical pickup unit-based displacement detection system using a specially designed holder with an installed air duct, in order to supply compressed air to the cantilever’s work area (Figure 6). The holder, with an installed 0.2 mm diameter air duct inside (Figure 2) was designed in accordance with two basic requirements: the duct must not interfere with the optical displacement measurement system; the air stream that flows from the air duct should create a maximum possible aerodynamic force on the upper surface of the cantilever. Additionally, an evaluation on the right size of micro tube was performed by theoretical research on the results of additional air flow simulation performed using FEM [16]. The distance between end of air duct and the upper surface of the cantilever was 0.4 mm.

For the precise supply of clean compressed air, a special mechatronic system was designed (Figure 4). The pressure of the compressed air in the system is controlled by changing the efficiency of the micro compressor according to the desired pressure and output pressure measured using the pressure sensor MPXV5050GP (Freescale Semiconductor, Austin, TX, USA).

For the control of the whole test rig, measurement system adjustment, calibration, and result processing, the LabView-based custom developed software was used. The control program allows for the simultaneous adjustment of the pressure of compressed air, and it records the displacement of the cantilever. Measurement data was recorded using the USB-6361 data acquisition instrument (National Instruments, Austin, TX, USA) at rates of up to 200 kSamples/s. During the experiment, compressed air was applied onto the upper side of the cantilever. In order to evaluate the effect of the sample surface, a piece of glass was placed at few micrometers under the probe. Air pressure was increased from 0 kPa to 20 kPa by step size 1 kPa, and the response of the cantilever was measured.

### 3.2. AFM Experiments

Cantilever’s vertical displacement at different applied pressures was measured by a home-made AFM setup using a modified AFM sensor with a cantilever, type NCHV from Bruker (Bremen, Germany) (Table 1, Case 1: stiff cantilever). The home-made AFM setup was composed using standard components from reliable producers. The research presented on this paper was performed using a home-made microscope, which requires access to the control algorithms and the ability to include modifications in the AFM structure. The main components of the AFM setup were: 3D-printed housing, AFM head based on a DVD optical pickup, manual micrometer translation stage, and closed-loop 3D nanopositioning stage (Nano-M350, Mad City Labs, WI, USA). The structure of the AFM setup is multi-level; first, the base is a mounted manual translation stage for rough positioning; on the top of this stage is a mounted nanopositioning stage Nano-M350 for precise positioning of the scanned sample. The nanopositioning stage have its own control system and this allows for precise positioning under a closed loop control. The nanopositioning XY travel range was 50 μm and 20 μm in the Z axis, with a resolution of 0.4 nm in the Z axis, and 0.1 nm in the XY axes. The nanopositioning stage was calibrated using internal sensors and linear coefficients according to the instructions provided by the manufacturer. An AFM head with attached cantilever and specially designed holder (Figure 4) is mounted on the top of AFM housing. A more detailed description of the home-made AFM setup is presented in [8]. During the experiment, the pressure of the applied compressed air stream was varied in the range 0–20 kPa at a pitch of 1 kPa, and the displacement (change in coordinate *η*_2_, Figure 1) of the cantilever was measured.

Horizontal scanning data, used to compare with the model output, was obtained by scanning the rectangular cross-section calibration grating APCS-0099 from Bruker (Bremen, Germany) with a height of 240 nm (2 µm pitch). Scanning was performed by a previously described home-made AFM with a cantilever, type NCHV from Bruker (Bremen, Germany) (Table 1, Case 1: stiff cantilever). Horizontal scanning was performed at 112.5 μm/s scanning speed in contact mode.

## 4. Results and Discussion

### 4.1. Experimental Results

**Figure 7:**
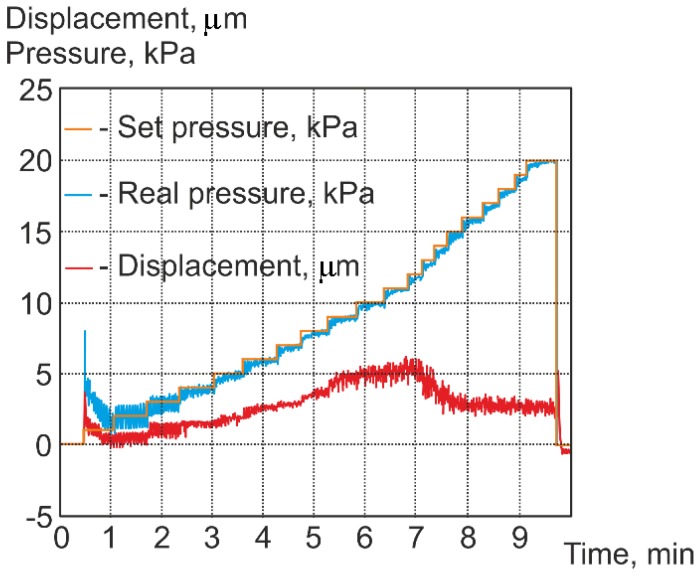
Experimental results of cantilever’s vertical displacement at different applied pressures.

The dependencies between the set pressure, real pressure, and cantilever displacement are shown in Figure 7. Set pressure is different from real pressure due to the properties of air supply and the control system of the stream pressure regulator. Air pressure in the system and cantilever displacement were measured continuously during the whole experiment. When the pressure of the compressed air was increasing from 0 to 12 kPa, the displacement of the cantilever increased linearly from 0 to 4 μm. When the pressure was increased above 12 kPa, the displacement of the cantilever decreased significantly. In this case, air flow created a lifting force between the cantilever’s lower surface and the sample surface, which compensated for the aerodynamic force (*F*_1_, Figure 1), exerted on the cantilever’s upper surface.

The performed experimental test of aerodynamic force implementation into the AFM cantilever control reveals a range of useful air pressures for our modified AFM measurement sensor as 1–12 kPa. The value of the supply pressure needed to be chosen regarding the aerodynamic losses that occur in the air supply system. Compressed air is supplied through a 4 mm diameter tube, connected through a 0.6 mm connector to a 0.2 mm air duct installed in a holder. The air duct with a small internal diameter 0.2 mm, was the main source of aerodynamic loses. However, implementation of an air duct with a bigger internal diameter is limited by the geometric properties of the cantilever displacement measurement system.

The relation between the real (measured) pressure and the effective pressure was detected indirectly, since experimental definition of the effective pressure from the duct was practically impossible. First, the FEM model of the air duct system, and the aerodynamic chamber between the duct end and the corresponding cantilever surface was solved [16]. The effective pressure of 60 Pa and necessary air flow rate of 1.946·10^−7^ kg/s were obtained indirectly from deflection of the AFM cantilever, and their values were defined from theoretical research of air stream parameters.

The experimental setup gave stable and permanent values of the air stream; therefore, to repeat this experimental research, required adjustment of the pneumatic duct to the particular design of AFM. Also, it is necessary to state that the calibration of the aerodynamic system was required after each change of the AFM sensor components.

The force exerted by the air stream influenced the dynamic properties of the AFM cantilever. Behavior of the solid body in the fluid stream was well researched, nevertheless, particular cases of some phenomena had to be accounted for. The behavior of the solid body in the stream depended on the flow regime, the stiffness of the solid body, and the aerodynamic properties of the body in the flow. A low velocity of flow stream with a laminar regime and a mode below the vortex generation acted like a nonlinear spring, with a coefficient of stiffness that was determined by the distance between the air duct and the surface of the AFM cantilever. When this distance was smaller than the diameter of the air duct, the air stream stopped acting as a spring. The proposed modernization of the sensor of AFM utilizes a laminar air stream flow regime with the flow parameters being far away from turbulent regime. Implementing an air stream into AFM increases the resonant frequency of the sensor, due to the additional stiffness that is applied to the sensor cantilever without changing its mass.

### 4.2. Analytical Results

The AFM dynamic system was simulated in two cases: with non-modified AFM sensor and modified AFM sensor models, using two types of cantilevers (Table 1).

**Figure 8:**
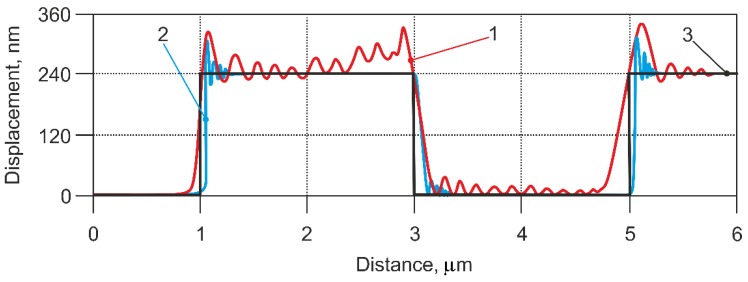
Comparison between the modelled and measured cantilever’s displacement, using a stiff cantilever (case 1 in the Table 1), and applying a scanning speed 112 µm/s. 1—experiment with non-modified AFM sensor; 2—response of non-modified AFM sensor from model; 3—theoretical profile of calibration grating.

Calculations for the AFM model in contact mode were first performed by simulating a scanning speed of 112 µm/s with a ‘stiff’ cantilever (Table 1, Case 1), and comparing this with the results of the scanning experiment (Figure 8). The experimental result fit quite well with the result from mathematical model, but in the results of the experiment, it was seen that some oscillations could have been caused by the inaccuracies of the calibration grating, or by other AFM components whose influences were not considered in the model.

**Figure 9:**
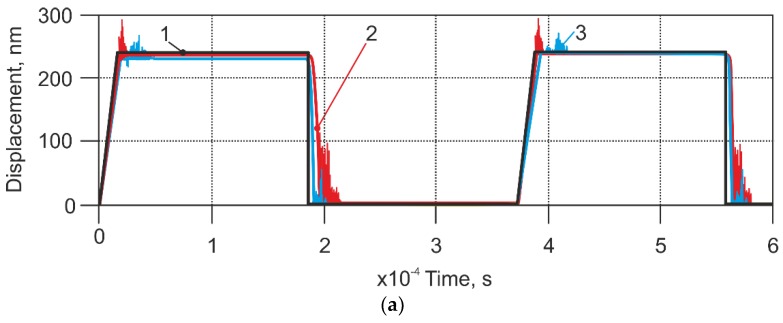

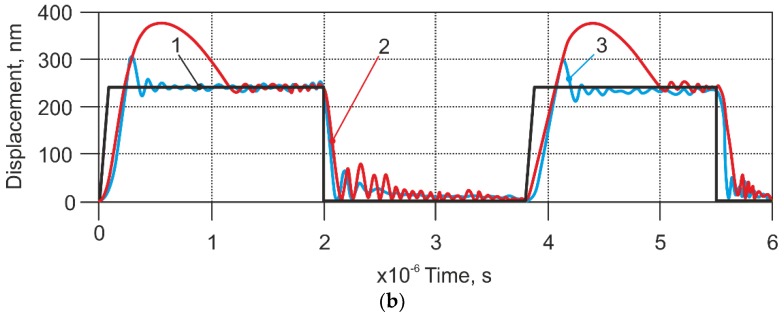
Modelling results of the displacement of the stiff cantilevers (case 1 in the Table 1). (**a**) Scanning speed 11.2 mm/s, (**b**) Scanning speed 1120 mm/s. Applied pressure: 60 Pa; 1—excitation signal; 2—response of non-modified AFM sensor; 3—response of modified sensor.

In further calculations we chose scanning speeds, which could cause contact loss between cantilever and sample surface in the model output. We observed contact loss at speeds of 11.2 mm/s (Figure 9a), and 1120 mm/s (Figure 9b). At a scanning speed of 11.2 mm/s, the contact loss was seen on the extremities of the surface topography and the bounce amplitude of cantilever tip was approximately twice as high (60 nm) compared to the response of the modified sensor (30 nm). The result of scanning output at 1120 mm/s speed is presented in Figure 9b). It was seen that when using a high scanning speed, the cantilever’s tip gave a very high displacement of about 370 nm, and the input was 240 nm. Moreover, analyzing the response of non-modified AFM sensors with respect to time, it was seen that approximately half of the “plateaus” were passed until the cantilever tip reached the sample surface. However, the sensor with applied pressure showed a response that was closer to the input signal. The cantilever bounce amplitude was about 300 nm, and the contact stabilization time was about five times shorter compared to the response of the non-modified sensor. Therefore, the difference between excitation and response for the non-modified sensor was 130 nm, while for the modified sensor it was 60 nm. Thus, in this case, vertical deviation from the scanned profile could be estimated as two times less than the usual AFM.

Modeling results of high scanning mode (1000 times higher than the standard speed) are presented in Figure 9b. In this case, the chaotic sample surface caused kinematic excitation of the cantilever with an average frequency that approached the resonant frequency of the AFM cantilever, which in our case was 320 kHz. The dynamic response had an oscillating character and ceasing of vibrations showed efficient damping. At the same time, the output of the modified AFM sensor remained intact, which we attributed to the effect of the applied air stream.

**Figure 10:**
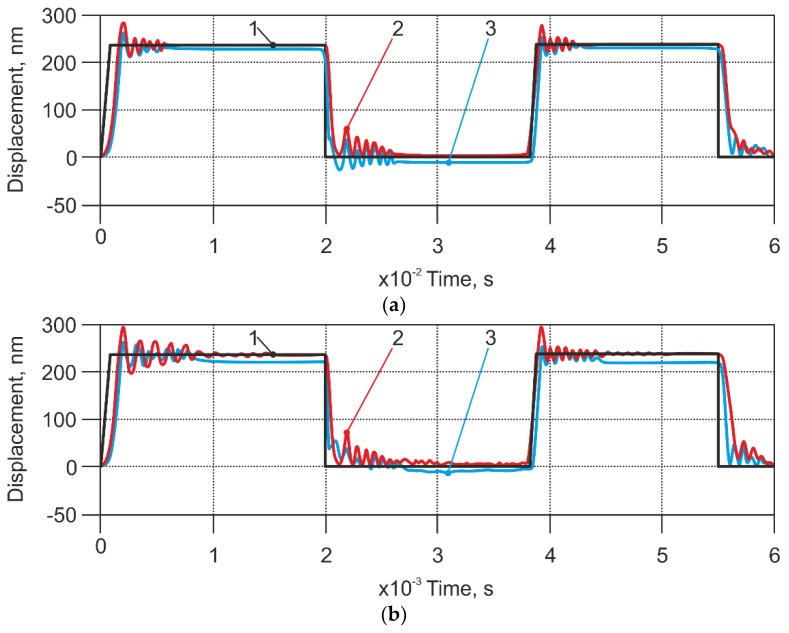
Modelling results of the displacement of the soft cantilevers (case 2 in Table 1). (**a**) Scanning speed 112 µm/s; (**b**) Scanning speed 1120 µm/s. Applied pressure 60 Pa. 1—excitation signal; 2—response of non-modified AFM sensor; 3—response of modified sensor.

Another set of results from the model in contact mode was obtained by using a ‘soft’ cantilever (Table 1, Case 2). First, the simulation experiment was performed at the velocity, which was not sensitive to the existing dynamic characteristics of the sensor (Figure 10). A modified sensor (with an applied air stream) gave a slightly lower signal than a standard sensor; therefore, there was no reason to apply any air stream.

The next simulation was performed at 10 times faster speed than the previous one, with the same pressure applied (Figure 10b). In this case, the modelling results of the standard and modified AFM sensor differed; the modified sensor produced lower vibration amplitudes and had a negative coordinate offset in comparison with the non-modified AFM sensor.

**Figure 11:**
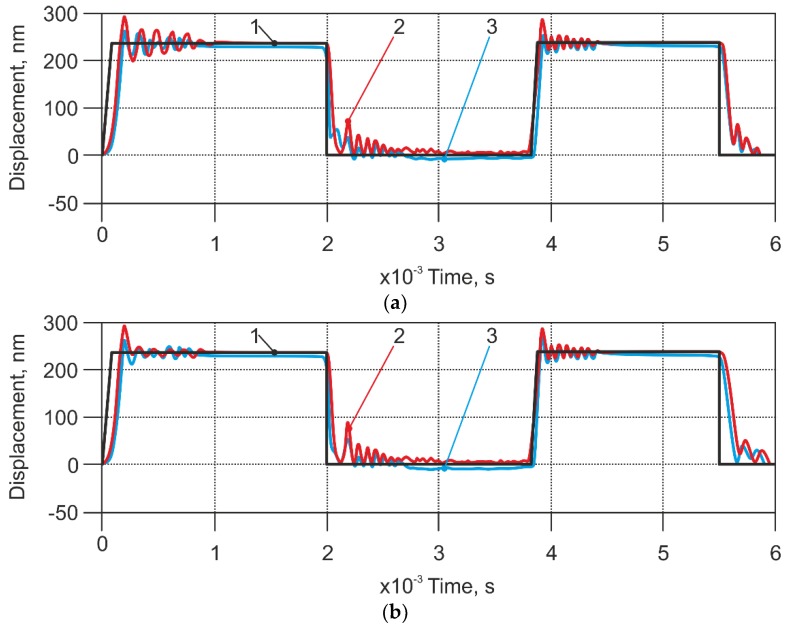
Modelling results of the displacement of the soft cantilevers (case 2 in the Table 1). (**a**) Applied pressure of 40.0 Pa. (**b**) Applied pressure of 20.0 Pa. Scanning speed of 1120 µm/s. 1—excitation signal; 2—response of the non-modified AFM sensor; 3—response of the modified sensor.

Our system with the applied pressure of 60 Pa gave an appropriate result; however, the modified sensor signal did not fit to the input signal. It was slightly lower, and this could be explained as the high pressure being applied, which disturbed the cantilever. Therefore, we tried to lower the pressure to 40 Pa and 20 Pa, as shown in Figure 11a,b, respectively. It was found that lower pressure was useful in the case of the softer cantilever. However, if the pressure was too low (20 Pa), its influence on measurement results for some lower values was limited.

Low pressure influence on the sensor signal revealed itself only on landscape extremities; nevertheless it can be useful for scanning relatively soft samples with sudden changes of surface height.

## 5. Conclusions

Implementation of the air stream as a nonlinear spring in the AFM sensor mechanical part demonstrated promising results: two times better vertical accuracy if compared with the unmodified AFM sensor working at 1120 mm/s (equivalent to 22 400 lines/s at 50 µm field of view) scanning speed, while the standard scanning speed of the particular AFM system is 112 µ/s (2.4 lines/s at 50 µm field of view). Our model output shows that by using the modified AFM sensor, there is a potential to improve the accuracy of the vertical axis as much as two-fold, if a stiff cantilever (spring constant of 40 N/m) is used. The efficiency of applying the non-linear additional stiffness depends on the stiffness and geometric parameters of the sensor cantilevers. The softer cantilever (spring constant of 0.2 N/m) in the case of the unmodified AFM sensor exhibits undesired oscillatory behavior already at 1.12 mm/s scanning speed, which provides an excitation that is close to the resonance frequency of the cantilever (which is 13 kHz). Our model shows that the signal can be improved by applying 20–40 Pa pressure. The same unmodified AFM sensor, if coupled with the stiff cantilever, will start to exhibit similar oscillatory behavior at 11.2 mm/s, for the same reason of excitation approaching the resonant frequency (320 kHz in this case). This undesired behavior can be eliminated by applying 60 Pa pressure. Modification of the sensor, by applying the air stream, creates the potential for an effective increase of the scanning speed of at least 10 times more, if a soft cantilever is used. Also, in the case of a stiff cantilever, the potential of the scanning speed improvement is at least 50-fold. We claim that our method to be efficient when the kinematic excitation, caused by the increased scanning speed, excites the oscillating behavior of the sensor.

Our model also shows that there is a potential for an effective increase of the scanning speed of up to 1120 mm/s; however, this is rarely practically possible in the physical AFM systems.

We also see some limitations and drawbacks for the proposed method. First of all, the effect of the non-linear force applied to the cantilever is limited when the lower values (more than 200 nm) are to be imaged. Second, the minimum pressure from the air stream is limited to 20 Pa in the case of the soft cantilever, to be effective. In the case of the stiff cantilever, this bottom limit is higher. Also, the maximum useful pressure is limited to 60 Pa in the case of the stiff cantilever, because the higher pressure will decrease the accuracy of the sensor. Correspondingly, for soft cantilevers, this upper limit will be lower, and its value definition requires further research.

The proposed dynamic model of the AFM sensor reproduces the experimental results well. Thus, it can be used to predict the scanning modes and to provide a scan parameter estimation for other kinds of modified AFM sensors.

## Figures and Tables

**Table 1:** Main parameters of modelled cantilevers.

Parameter	Case 1: Stiff Cantilever	Case 2: Soft Cantilever
Constant, *α*	0.0009	0.0009
Length, *L*	117 μm	450 μm
Mass, *m*_1_	5.31 × 10^−11^ kg	9.53 × 10^−11^ kg
Mass, *m*_2_	2.65 × 10^−11^ kg	4.77 × 10^−11^ kg
Resonant Frequency, *f*	320 kHz	13 kHz
Size of the initial gap, Δ_0_	0.4 mm	0.4 mm
Diameter of air duct	0.2 mm	0.2 mm
Spring Constant, *k*	40 N/m	0.2 N/m
Thickness, *t*	3.5 μm	2 μm
Width, *w*	33 μm	50 μm
Young’s modulus, *E*	310 GPa	310 GPa
Manufacturer	Bruker	NanoWorld Services
Type	NCHV	CONTR

**Figure A1:**
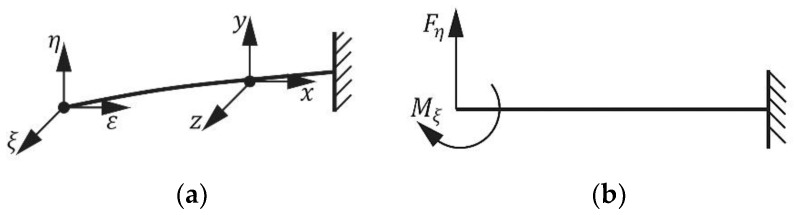
(**a**) Local coordinates of the beam for potential energy evaluation; (**b**) Local generalized coordinates of the beam.

## Appendix A

Kinetic energy of the system:

(A1) $$T=\frac{1}{2}\left(m_{1}{\dot{\eta }}_{1}^{2}+I_{1}{\dot{\gamma }}_{1}^{2}+m_{2}{\dot{\eta }}_{2}^{2}+I_{2}{\dot{\gamma }}_{2}^{2}\right).$$

The matrix of inertia is given as:

(A2) $$\left[A\right]=\left|\begin{array}{cccc} m_{1} & 0 & 0 & 0\\ 0 & I_{1} & 0 & 0\\ 0 & 0 & m_{2} & 0\\ 0 & 0 & 0 & I_{2} \end{array}\right|.$$

The potential energy is found using the methodology [18] as shown in Figure A1a.

It is considered that in its natural state, the rod is rectilinear and bending occurs only in one plane, as illustrated in Figure A1b. In this case, the potential energy of the beam is:

(A3) $$\Pi =\frac{1}{2}\int_{0}^{L}\frac{{\left(M_{\xi }-sF_{\eta }\right)}^{2}}{EI_{sk}}ds,$$

where *L*—length of the beam; *s*—local longitudinal coordinate of the beam.

In this case, the local generalized coordinates of the beam are force *F_η_* and the bending moment *M_ξ_*. By differentiating the potential energy of the beam with respect to the generalized coordinates *F_η_* and *M_ξ_*, the matrix of flexibility is obtained:

(A4) $$\left[\beta \right]=\left[\begin{array}{cc} \frac{L^{3}}{3EI_{sk}} & -\frac{L^{2}}{2EI_{sk}}\\ -\frac{L^{2}}{2EI_{sk}} & \frac{L}{EI_{sk}} \end{array}\right].$$

The matrix of stiffness:

(A5) $$\left[C_{stiff}\right]={\left[\beta \right]}^{-1}=\left[\begin{array}{cc} \frac{12EI_{sk}}{L^{3}} & \frac{6EI_{sk}}{L^{2}}\\ \frac{6EI_{sk}}{L^{2}} & \frac{4EI_{sk}}{L} \end{array}\right].$$

Because the cantilever of the AFM system is modelled by two elements, the *i*th element of the matrix of stiffness is:

(A6) $$\left[C_{stiff_{i}}\right]=\left[\begin{array}{cc} \frac{12EI_{sk}}{L_{i}^{3}} & \frac{6EI_{sk}}{L_{i}^{2}}\\ \frac{6EI_{sk}}{L_{i}^{2}} & \frac{4EI_{sk}}{L_{i}} \end{array}\right].$$

In our case, the cross-section of the beams and the Young’s modulus are equal for both elements. The potential energy of the cantilever is given by:

(A7) $$\Pi_{Can}=\sum_{i=1}^{2}\Pi_{i}=\Pi_{1}+\Pi_{2},$$

where *π_i_*—potential energy of *i*th beam of the cantilever:

(A8) $$\Pi_{1}=\frac{1}{2}\left[\eta_{1};\gamma_{1}\right]\left[C_{stiff_{1}}\right]\left\{\begin{array}{l} \eta_{1}\\ \gamma_{1} \end{array}\right\},$$

(A9) $$\Pi_{2}=\frac{1}{2}\left[\left(\eta_{2}-\eta_{1}\right);\left(\gamma_{2}-\gamma_{1}\right)\right]\left[C_{stiff_{2}}\right]\left\{\begin{array}{l} \eta_{2}-\eta_{1}\\ \gamma_{2}-\gamma_{1} \end{array}\right\}.$$

Total potential energy of the mechanical sensor of AFM:

(A10) $$\Pi =\Pi_{Can}+\Pi_{3},$$

where Π_3_—potential energy of the AFM sensor stiffness:

(A11) $$\Pi_{3}=\frac{1}{2}k{\left(\eta_{2}-\delta \right)}^{2}.$$

The coefficient of stiffness *k* is piece-wise linear. To evaluate the processes that occur during the calculations: when the spring is compressed, *k* = *k*_0_; when the spring is uncompressed, *k* = 0. This way, two different states of the system are obtained. The coefficient of stiffness of the one system state is obtained by differentiating potential energy Π with respect to the generalized coordinates *η*_1_, *γ*_1_, *η*_2_, *γ*_2_.

(A12) $$\left[C\right]=\left|\begin{array}{cccc} \frac{24EI_{sk}}{L_{1}^{3}}+\frac{24EI_{sk}}{L_{2}^{3}} & \frac{12EI_{sk}}{L_{1}^{2}}+\frac{12EI_{sk}}{L_{2}^{2}} & -\frac{24EI_{sk}}{L_{2}^{3}} & -\frac{12EI_{sk}}{L_{2}^{2}}\\ \frac{12EI_{sk}}{L_{1}^{2}}+\frac{12EI_{sk}}{L_{2}^{2}} & \frac{8EI_{sk}}{L_{1}}+\frac{8EI_{sk}}{L_{2}} & -\frac{12EI_{sk}}{L_{2}^{2}} & -\frac{8EI_{sk}}{L_{2}}\\ -\frac{24EI_{sk}}{L_{2}^{3}} & -\frac{12EI_{sk}}{L_{2}^{2}} & k+\frac{24EI_{sk}}{L_{2}^{3}} & \frac{12EI_{sk}}{L_{2}^{2}}\\ -\frac{12EI_{sk}}{L_{2}^{2}} & -\frac{8EI_{sk}}{L_{2}^{2}} & \frac{12EI_{sk}}{L_{2}^{2}} & \frac{8EI_{sk}}{L_{2}} \end{array}\right|.$$

The matrix of stiffness for other states of the system differ from the previous state, only that here, *k* = 0. It is considered that damping matrix is:

(A13) [B]=α[C],

where α—constant, and in our case, its value is 0.0009.

The α value is determined from the results of experimental research and has no definite physical meaning. Such a method of proportional damping of the stiffness is often used in systems with an experimentally defined general coefficient of stiffness.

Finally, the vector of generalized forces is:

(A14) $$\left\{Q\right\}=\left\{\begin{array}{c} -F_{1}\\ 0\\ -F_{2}+k\delta +h\dot{\delta }\\ 0 \end{array}\right\}.$$
